# Exploring Barriers and Facilitators of Inter-Organizational Management in Response to Mass Casualty Traffic Incidents: A Qualitative Study

**DOI:** 10.30476/BEAT.2021.89416

**Published:** 2021-04

**Authors:** Seyed Javad Sadat, Ardashir Afrasiabifar, Davoud Khorasani-Zavarehg, Mohamad Javad Moradian, Mohammadreza Vafaeenasab, Abbasali Dehghani Tafti, Hossein Fallahzadeh, Mahsa Khodayarian

**Affiliations:** 1 *Department of Health in Emergencies and Disasters, School of Public Health, Shahid Sadoughi University of Medical Sciences, Yazd, Iran*; 2 *School of Nursing, Yasuj University of Medical Sciences, Yasuj, Iran *; 3 *Skull Base Research Center, Loghman Hakim Hospital; Department of Health in Emergencies and Disasters, School of Public Health and Safety, Shahid Beheshti University of Medical Sciences, Tehran, Iran*; 4 *Trauma Research Center, Shahid Rajaee (Emtiaz) Trauma Hospital, Shiraz University of Medical Sciences, Shiraz, Iran*; 5 *Yazd Accident Prevention and Crisis Research Center, Shahid Sadoughi university of Medical Sciences, Yazd, lran*; 6 *Healthcare Data Modeling Center, Departments of biostatistics and Epidemiology, School of public health, Shahid Sadoughi University of Medical Sciences, Yazd, Iran*

**Keywords:** Inter-organizational management, Mass casualty, Traffic incidents

## Abstract

**Objective:**

To investigate and understand the current status of inter-organizational management in relief organizations as well as the relief organizations personnel behavior when facing mass traffic incidents (MCTI). The inter-organizational barriers and facilitators are also discussed in response to MCTI management and in order to help direct future actions to improve pre-hospital emergency services.

**Methods:**

The current qualitative study was performed through face-to-face, semi-structured interviews with 31 individuals from pre-hospital emergency services authorities and personnel, Red Crescent and Yazd, Kohgiluyeh and Boyer-Ahmad, Fars, and Qom provinces police. These provinces were selected by purposive sampling in 2018-2019. The conventional content analysis method was applied to analyze the data in this research.

**Results:**

Three main categories and 14 subcategories were determined. The categories are including relief organizations coordination (having four subcategories: independent relief organizations, interdepartmental services integration, insufficient knowledge of organizations about one other, and performance based on job descriptions), resource and infrastructure management (having four subcategories: adverse information management, proper information management, lack of medical resources and capacities considered, and upgrading of medical resources and capacities considered), and response management of relief organizations (having six subcategories: incomplete assessment, improving the quality of assessment, weakness in establishing scene security, scene security management, poor response, and cooperation in response).

**Conclusion:**

Relief organizations need to perform under a unified command. It has inter-organizational cooperation and provide integration of interdepartmental services in order to manage responsiveness at the scene. It also prevents an independent, chaos, and inability of the injured to properly understand and needs in MCTI.

## Introduction

Mass casualty incidents (MCI) are those incidents that cannot be managed by using present facilities, resources, and an usual operational organizations processes during pre-hospital emergency medical services [1]. A traffic incident with at least three deaths or five injuries is considered an MCI according to the medical emergency personnel [2]. Traffic incidents are one of the leading causes of death throughout the world especially in low and middle income countries [3] including Iran [4]; highlighting the necessity of emergency medical services management. Pre-hospital emergency medical services are the foundation for MCTI management [5], which emphasizes the effectiveness of traffic incidents management [6]. Some organizations are involving to manage traffic incidents such as pre-hospital emergency medical services, Red Crescent, police, and fire departments. Relief organizations’ performance are including staff dispatch to the incident location and providing quick services to the injured [7]. Before the incident, all of these measures need policy making and planning [8]. In addition, relief organizations cooperation is necessary according to their job description, since lack of awareness of other relief organizations disrupts inter-organizational cooperation of identities and roles [9]. Sometimes, special organizational objectives make the relief organizations unresponsive to the incidents and leads to their non-cooperation [10]. Therefore, relief organizations need to have a common language, promote inter-organizational cooperation and develop communication networks to achieve successful performance in MCTI [11]. 

In casualties caring, past experiences have shown that organizing the staff and optimizing resources reduce fatalities by 8-50% [12]. This reduction needs professional interaction of relief organizations with a common command during an incident, therefore, caring for the most injured individuals can be provided [13]. However, Hitti *et al*., [14] concluded that relief organizations lack a developed plan for responding to MCI and their personnel are not aware of their roles and responsibilities. They also suggest that relief organizations are familiarizing with their personnel duties response through practicing of how to respond to an MCI.

The incidents’ number rise and society’s vulnerability have increased the demand for pre-hospital emergency services [15]. Based on this fact, the relief organizations response to the increasing needs of societies must be studied in order to help reaching an evidence-based management [8]. A research has been done with a quantitative approach on the factors influencing incident management and ways to improve performance in MCTI. However, qualitative studies provide an in-depth understanding of concepts and phenomena [16]. In order to guide future efforts to improve pre-hospital emergency services, the current study has been designed based on relief organizational managers and personnel experiences to assess and understand the current status of relief organizations management, the relief organizations’ personnel behavior when dealing with MCI, and ways to manage the MCTI response.

## Materials and Methods

*Approach*

A qualitative research method was applied with the conventional content analysis approach, since the current study aimed is to explore the factors affecting management of relief organizations in pre-hospital MCTI services. The conventional content analysis is a research method used for analyzing and interpreting the data in qualitative studies. In this method, analyzing textual data were performed and themes or explicit and hidden patterns were recognized through an orderly categorization in the text [17]. Such incident’s management were complicated by considering the fact that various organizations were involved to MCTI respond, as inter-organizational coordination and resource allocation are needed [18]. Therefore, the qualitative research method was applied with the aim of describing the underlying phenomena and providing new knowledge, insight, and practical guidance in this study [17]. 

*Setting*

The current study was conducted through in-depth interviews about the experiences of the beneficiary in Yasouj, Yazd, Shiraz and Qom from June 2018 to September 2019. Upon prior coordination, interviews were carried out by individuals who had theoretical knowledge or practical experience in MCTI relief of inter-organizational management. Organizations include relief personnel in pre-hospital emergency centers and sites, police department and Red Crescent.

*Participant Selection*

Thirty-one individuals were included in the study of which, 9 was emergency medical technicians, 5 emergency medical experts, 3 emergency operation center (EOC) officials, 3 dispatch operators, 3 medical emergency management centers heads, 3 police officers, 2 Red Crescent relief workers, 1 Red Crescent relief and rescue deputy, 1 emergency medicine specialist and 1 road maintenance official. Participants were selected through purposive sampling. The study inclusion criteria were to have at least 2 years work experience, conscious interest to participate in the interviews and the ability to express experiences. 

*Data Generation*

The data collection method initially included an unstructured interview following by a semi-structured interview with open-ended questions. The ﬁrst four interviews were carried out by unstructured in-depth interviews following 27 semi-structured interviews. The main interview questions were: “explain your experiences with an MCTI attendance”, “explain the factors affecting MCTI”, describe a clear understanding of the information provided by the participants, ask exploratory questions e.g. “what do you mean?”, “please explain”, and “provide an actual example of your experience”. Finally, the interview was finished with a question as there was anything missing that participants’ thought should be raised or they wanted to speak more. The interviews duration varied between 25-110 minutes. The interviews were recorded by using mobile audio recording software in a quiet and pleasant environment. Upon interview, the recorded interviews were transcribed as word-for-word textual data. Reminder writing was also used during the data collection. All the interviews were transcribed and typed by using the Microsoft Office 2013 software in the day after interview. 

*Data Analysis*

The Elo and Kyngas’ analysis method was applied in the current study. This method involves open coding, creating categories and abstraction [19]. The analysis was carried out immediately after the interviews transcription. To do this, the interviews were first listened several times in order to gain a general understanding of the interviews. Then, the interviews were transcribed and precisely studied several times. Codes were extracted and categorized based on similarities and differences. The resulting categories were placed in the main categories based on the meanings and concepts commonalities. In the next step, the categories were compared and themes were emerged through analysis and interpretation of the main categories. The researcher “stayed close to the data” and attempted to set aside her own beliefs, thoughts and pre-contemplated ideas about phenomena studied in coding and analysis of the responses; a process known as “bracketing”. The authors of the current study followed Consolidated Criteria for Reporting Qualitative Research (COREQ) to report the results.

*Ethical Considerations*

The current study has been approved by Yazd Shahid Sadoughi University of Medical Sciences with the ethics code of IR.SSU.SPH.REC.1397.020. Permission was granted to conduct the interview by receiving an introduction letter from the Faculty of Health and presenting it to the Medical Emergency and Accident Management Center Universities of Medical Sciences in Yazd, Shiraz, Kohgiluyeh and Boyer-Ahmad and Qom provinces. Written consent was obtained from participants for their participation in the study. The participants were notified for an interview confidentiality and their right to withdraw from the study at any time and in any part of the study. The interview time was decided at the participants’ will. 

*Rigor*

In order to validate the data, Lincoln and Guba’s criteria were used [20]. Prolonged researcher engagement, sufficient participation, participant’s appropriate interaction and data verification were checked with credibility of the data. Dependability or reliability of the findings was achieved by external checking, the codes recurrent review, categories, and subcategories. The sampling diversity and time triangulation were increased credibility and data confirmability in participants. The interviews text, codes and the obtained categories were confirmed through peer checking by two faculty members. Transferability or fittingness was made possible by presenting participants’ statements to the rich data explanation and scientific consultations from expert professors. 

## Results

The participants’ average age was 38 years old and the average work experience was 13 years (Table 1). Three main categories and 14 subcategories were determined (Table 2).

**Table 1:** Demographic characteristics of the participants

**%**	**NO (n=27) **		
90%	28	Male	Sex
10%	3	Female	
16%	5	25-30	Age
35%	11	31-35	
23%	7	36-40	
26%	8	Over 40	
6%	2	2-5	Experience (years)
26%	8	6-10	
68%	21	Over 10	
16%	5	Associate’s degree	Education
48%	15	Bachelor’s degree	
23%	7	Master’s degree	
3%	1	General physician	
10%	3	Post Doc	

**Table 2:** Barriers and facilitators of inter-organizational MCTI management by relief organizations

**Example of codes**	**Subcategory: Facilitators**	**Example of codes**	**Sub category: Barriers**	**Category**
Inter-organizational planning	Integration of Interdepartmental Services	Islanding operation of organizations	Independent Relief Organizations	Coordination of Relief Organizations
Inter-organizational training of relief organizations		The gap between the plan and operation		
Division of work program	Performance based on Job descriptions	Contradiction in duty description	Insufficient Knowledge of Organizations about one other	
Assigning a person responsible for the dead		Miscomprehension of mutual limitations		
Receiving complete information	Proper Information management	Inadequate communication equipment	Adverse Information Management	Resource and Infrastructure Management
Centralized bases of relief organizations		Incomplete information sharing		
The need to update scientific information and equipment	Upgrading of Medical Resources and Capacities	Hospital’s defective services	Lack of Medical Resources and Capacities	
Need to increase equipment in MCI		Inadequate knowledge of hospital personnel		
Need to carefully assess the injured	Improving the Quality of Assessment	Disruption of people in scene assessment	Incomplete assessment	Response management of relief organizations
The necessity of triage training		Neglect of triage		
Determining a safe place to provide services	Scene Security Management	Unstable security of the scene	Weakness in Establishing Scene Security	
Control and management of people’s behavior		Secondary incident		
Leadership of an organization	Cooperation in response	Personnel disorder	Poor response	
Cooperation of relief organizations’ personnel		Lack of concurrent presence of relief organizations		

*Relief Organizations Coordination*

In some cases, and according to the participants’ experiences, the relief organizations’ personnel arrived at the scene with delay. In case of timely arrival, they did not have the required coordination to provide services for injured peoples. A unified command lack and managerial coherence absence have led to chaos and irresponsible interferences in one another’s roles. The MCTI relief organizations’ managers did not have the necessary cooperation to provide optimal services for the injured people. Instead, they were provided services regardless their own responsibility which sometimes it will cause irreparable damage to the injured people. This category includes four subcategories such as an Independent Relief Organizations, Insufficient Knowledge of organizations about one other as Obstacles, Interdepartmental Services Integration and Performance Based on Job Descriptions as Facilitators. 

*Independent Relief Organizations*

In response to rescue operation, relief organizations should play their roles according to the duty descriptions and as a team. However, they lacked teamwork and each one acted regardless of what is needed at the scene based on their duty description. They did not have a specific operation plan; even if there was a plan, it would not be implemented. When cooperation needed, the relief organizations personnel were focused on organizational details regardless of the service system comprehensiveness. *“a crowded scene leads to lack of coordination and lack of cooperation from a relief organization causes lack of coordination; an organization may say that they will be acting independently.” (EMT3)*

*Interdepartmental Services Integration*

The participants stated that having a joint communication center, centralized relief bases and joint relief exercises would be an effective force in the cohesion of services provided. They believed that all the relief organizations should follow a unified procedure at the scene while maintaining their independence; therefore, the incident scene is managing well. *“If we have a joint dispatch or joint bases, it will be effective even in managing the incident scene.” (EMT2)*

*Insufficient Knowledge of Organizations about one Other*

Relief organizations have independent bases and provide services based on the targets at various spatial and temporal distances. Normally, an organization does not have close relationship with and sufficient knowledge of services, performance, limitations and another organization potential. Such adequate knowledge requires constant relationship between the organizations for providing more quality services. *“We do not normally have contact with police but in MCI we have to be together. We must know the redlines of each other. Police must know my triage but they do not. At normal times, we have no work relationships with the Red Crescent but they should know the techniques of our job and I need to have at least basic information about relief and rescue. “(Head of Pre-Hospital Emergency)*

*Job Descriptions Performance*

Providing services of other relief organizations were among the facilitators and accelerators in providing services by relief organizations based on duty descriptions and avoiding duties interference. In addition to performing their duties, firefighters were smoothed the conditions for pre-hospital emergency personnel; therefore, they can provide services. They also advised the people at the scene against interfering with treatment of the injured people.* “Before we arrived, the firefighters at the scene had opened a separate way for the ambulance to enter the scene and kept the way open for other ambulances to arrive. They told people at the scene not to touch the injured people and allow the emergency colleagues to take the necessary measures.” (EMT8)*

*Resource and Infrastructure Management*

The present study findings show that there are no proper communication resources and infrastructure, the information exchange of the injured and the needs of the scene with the dispatch center is not possible. Lack of human resources and medical equipment has led to the lack of timely relief and medical services to the injured whom has caused irreparable damage. This category has four subcategories which considered as facilitators includes information management adverse, lack of medical resources and capacities considered as barriers, information management, and upgrading of medical resources and capacities.

*Adverse Information Management*

The dispatcher transmits the scene information to the personnel before the relief organizations arrive. According to the participants and in some cases, the information received by the dispatcher were incomplete which relief organizations send fewer pieces of equipment, facilities, and even relief personnel to the scene. *“The emergency operator did not receive complete information whether there was anybody trapped in the crashed car? How many injured? When we arrived at the scene, we found two people dead and two more stuck in the car and we had not taken a rescue vehicle with us. It took a quarter of an hour for the rescue vehicle to arrive. On the other hand, another injured person passed away while waiting for the rescue equipment because we had no information about the injured.” (Red Crescent deputy of relief and rescue)*

*Proper Information Management*

The dispatcher is responsible to provide the information of the incident scene to the relief organization personnel. Upon receiving correct and comprehensive information about the incident scene, most of the participants in this study emphasizes that the relief organizations will be called timely and this will accelerate management of the scene, treatment, and transferring the injured people. *“Due to the our message center specifications, it coordinates with the Red Crescent for rescuing the injured people who may have been possibly trapped in a car; or it may coordinate with the fire department when a car explosion is possible; or in case of any fight at the scene, it coordinates with police in advance.” (EMT2)*

*Medical Resources and Capacities Lack*

At the time of MCTI, the resources of relief organizations and the hospital and pre-hospital therapeutic capacities did not meet the needs of the injured people at the incident scene and in the hospital. The personnel did not have necessary and quality equipment. The reception of the injured people at the trauma center happened with delay. Sometimes, the emergency medical equipment replacing was not possible and the capacity of medical hospital was not enough to admit those injured in mass-casualty incident (MCI). *“With a high number of injured people, the trauma center cannot admit them; they do not have enough beds available. We did not have consumer equipment. Unfortunately, the hospital had a shortage equipment and they did not replace them quickly; for example, they did not have alternative splints.” (EMT6)*

*Medical Resources and Capacities Upgrade*

According to the participants’ statement, equipping relief organizations with relief equipment and equipment training is a necessity that makes the first manpower present at the scene to be able to identify and perform the necessary relief services. Accident equipment, equipped ambulance, quality equipment and increased knowledge of its use, and facilitate services were located at the accident scene. *“Ambulance, the personnel equipment and their science is changing day by day worldly. We must stay up to date. If our equipment is outdated, relief is practically not what we want.” (EMT 2)*

*Response Management of Relief Organizations*

Based on the participants experiences, the relief services’ call and management at the accident scene are subject to receive accurate information and the relief organizations cooperation in responding at the scene of the accident. If the security of the scene has not provided due to delays and shortages of security forces, the personnel of relief organizations manage the scene of the accident. Also, provide relief services will face challenges that will weaken the response of relief organizations. Holding training workshops and joint relief exercises will improve the level of assessment, security and response management of relief organizations. Response management with its six subcategories includes an incomplete assessment, weakness in establishing scene security, poor response as barriers, improving the quality of assessment, scene security management and cooperation in response as facilitators.

*Incomplete Assessment*

The findings of this study showed that overcrowding and the peoples presence and gathering at the scene has disrupted the services of relief organizations. A large number of injuries, the high workload and the small number of personnel in its first ambulance contribute to an environment for making poor decisions. Overcrowding and in some cases unwarranted interference have prevented accurate assessments and resources at the scene. *“In some cases, we have to walk hard among the people and reach the injured. This makes it difficult for us to assess the injured and we cannot assist the injured. Conditions are not proper and overcrowding makes it worse” (EMT23)*

*Improving the Assessment Quality*

According to the participants, the information from the personnel of the relief organizations is limited to their organizational activities. They are not able to assess the scene and evaluate the injured when they are present at the scene of the accident before the other personnel of the relief organizations. The need to train personal is an inevitable necessity by using the equipment, how to provide services, the other organizations equipment use by holding training programs and joint operational exercises will improve the service’s quality. *“Police and relief forces are present at the scene of the accident, they cannot perform triage, and they must receive the necessary training in this field; therefore, they can perform triage and control the scene before the emergency forces arrive.” (EMT3)*

*Weakness in Establishing Scene Security*

The most of the participants consider the necessary scene securing to provide services to the injured by the relief organizations. Failing to secure the scene can cause secondary incidents with irreparable damages even worse than those already happen to the injured at the scene. *“The traffic police guided the road and tried to minimize the number of vehicles at the scene. Because they did not have enough officers, they could not fully respond and manage properly which led to a secondary incident.” (EMT23)*

*Scene Security Management*

In some cases, the scene has been well secured. In some incidents, police could control and secure the scene with the timely arrival, lighter traffic of personal vehicles and smaller crowd at the scene. Even darkness was not a barrier for the scene security. *“Although the incident happened at night, the security of the scene was ensured very well. The location of the incident was close to a police station and it was night with fewer people passing by.” (EOC authority)*

*Poor response*

The MCTI personnel of relief organizations at the scene cope with problems such as delayed arrival of the personnel, shortage of medical and release facilities and equipment, insecurity of the scene, and poor services to the injured people. The relief organization personnel were beaten to secure the scene because of failing. They did not have the release equipment suitable for the scene. They transferred the red-flagged injured people to the ambulance bus, instead of the yellow-flagged ones. *“An operation colleague was beaten at the scene. The security of the scene had not been ensured. When we arrived at the scene, we needed to do release operations but the Red Crescent did not have the release equipment. My colleagues transferred two red-flagged injured people to the ambulance bus while they were supposed to be transferred with an ambulance immediately.” (Red Crescent deputy of relief and rescue)*

*Cooperation in Response*

One of the issues raised by the participants in the interviews was that relief organizations should benefit from each other’s capacities. Enhancing incident response capabilities involves equipment and training efficient personnel. Inter-organizational training includes theoretical and practical training for optimal, effective responsiveness. *“we should move toward agility; the personnel of all the organizations should pass inter-organizational courses and should learn how to use the equipment of other organizations. The police forces should know how to perform CPR.” (EOC authority)*

## Discussion

The purpose of this study was to explain the facilitators and barriers to the management of relief organizations in response to MCTI. The results of the study showed that the key factors in the inter-departmental management of relief organizations were the coordination of relief organizations, the resource and infrastructure management and the response management of relief organizations. Each of these factors was influenced by barriers and other factors as facilitators. Barriers to the coordination of relief organizations were the independence of these organizations and having insufficient knowledge of each other. The facilitators were unified inter-organizational services and performance based on their duty descriptions. Barriers to resources and infrastructure management were Adverse Information Management, lack of medical resources and capacities. Facilitators were proper information management and upgrading of medical resources and capacities. Incomplete assessment, weakness in establishing scene security and poor response were the barriers to managing the response of relief organizations. improving the quality of assessment, scene security management and cooperation in response were the facilitators. Interdepartmental management in MCTI, in addition to improving service quality and reducing losses, has led to the optimal use of available resources. Pre-hospital services in Iran are managed by several organizations such as the Medical Emergency and Accident Management Center, the Red Crescent, the police and the fire department, which has led to disruptions in related activities. Studies of Pre-hospital emergency organizations are integrated and independent in developed countries. The most important feature of emergency systems in developed countries are the single management of pre-hospital and in-hospital emergency services and online medical guidance. In Iran, prehospital emergency systems do not have such features [21]. 

According to the findings of this study, the lack of a centralized command system makes it a challenge to coordinate between organizations and to use all capacities for managing events efficiently. Negative competition and conflict of interest between relief organizations are evident at the scene. Relief organizations did not have a specific plan for coordinating and managing the scene. The presence of multiple commanders, each of whom acted independently based on a description of organizational duties, challenged incident management. In line with the above findings, other studies have reported the presence of multiple commanders and the issuance of conflicting orders at the scene of the accident as a cause of confusion and inconsistency of relief organizations and recommend joint planning and obedience of all relief organizations to a single command to coordinate and efficient operations [6, 8]. The results of other studies have also reported the lack of unit command in Relief organizations as one of the management problems in mass-traffic incidences (MCTI) [8]. One of the emphasized factors that inhibit pre-hospital services is the autonomy and independence of relief organizations [22]. 

In this study, one of the obstacles to the coordination of relief organizations was the lack of familiarity between relief organizations. Due to unfamiliarity with the services provided by other organizations at the scene of the accident, organizations do not have a correct understanding of each other’s services, and this provided a condition for non-cooperation at the scene of the accident. In addition, different organizational structure and goals in accidents was another reason for the non-cooperation of relief organizations. There is no proper interaction and communication between Relief organizations. Lack of communication and interaction between organizations has led to insufficient knowledge of the duties, resources and services of other organizations, which has led to incoherence and inefficiency of relief operations and as a result, disruption in providing services to the injured. In confirmation of the above findings, the results of other studies have attributed the lack of coordination of Relief organizations due to insufficient knowledge of Relief organizations of each other’s duties [23, 24]. In this regard, studies have shown that the complexity of organizational structures and different organizational goals have led to the non-cooperation of organizations [25, 26].

The integration of interdepartmental services and task-based performance were facilitators of coordination for relief organizations. Integrating command and improving the coordination of relief organizations at the scene of an accident improves service delivery for the injured and reduces the likelihood of delays and deaths [26]. The main challenge for incident commanders during joint response operations is to coordinate the participating actors and their available resources. This coordination must consider the various elements of organizations, their structures, roles and tasks in order to be effective. Whether these elements are contradictory or not will affect the effectiveness of the response operation [27]. Therefore, planning and coordination of police, relief and rescue and EMS is a key step in managing accidents with mass injuries [28]. Also, according to the results of existing studies, the most important management component in preventing deaths based on traffic accidents is the coordination of external and internal Relief organizations [29]. Division of duties, active cooperation of Relief organizations in the management of the injured and their proper communication at the scene of the accident is one of the effective components in improving the quality of pre-hospital care [30]. Clear responsibility and coordination of Relief organizations lead to coherence in service, which is an inevitable necessity in the performance of Relief organizations personnel [31]. Another finding of this study was resource and infrastructure management. Disruption of the emergency center’s radio and communication system with the accident scene, incomplete receipt of information and incomplete receipt of accident scene information by the dispatch user caused Relief organizations to arrive at the scene of the accident with a delay. As a result, this issue has disrupted the process of scene management and cooperation of Relief organizations and disrupted services to the injured. Numerous studies have reported communication problems as one of the most serious and important challenges of MCTI [8, 9]. The most important communication problems have been reported as technical disturbances in mobile networks and radio channels on the route or at the scene of the accident [8, 32], failure of communication equipment and infrastructure, leading to inability to contact personnel, lack of tracking units at the scene and lack of information from the security of the accident[8, 9]. Incomplete information has been one of the obstacles to Relief organizations’ cooperation. Lack of communication and lack of information sharing leads to disruption of Relief organizations and the disruption of their services [32]. This study showed that providing communication infrastructure and providing complete and accurate information to relief organizations leads to the cooperation of relief organizations at the scene and the effectiveness of their services. In this regard, other studies have emphasized the effective role of information technology. It provides accurate scene information to relief organizations in order to assist in making correct decisions and cooperation [32, 33]. As the participants stated, one of the most effective factors in improving management is information management and information sharing to coordinate relief organizations. Lack of a common contact number, poor communication between organizations, lack of information sharing, lack of a single command are some of the key obstacles to managing relief organizations. Sharing information between different organizations strengthens the cooperation of organizations and the rapid deployment of personnel at the scene and facilitates the execution of relief services [34]. Lack of resources and therapeutic capacity was a barrier to management in this study. Insufficient staff, lack of resuscitation equipment at the scene of the accident has delayed the provision of medical services. Other studies have also considered the lack of manpower, physical facilities and equipment of the pre-hospital and hospital system as the cause of disruption in providing services to the injured [29, 30]. The main obstacles to providing emergency medical services are lack of resources and poor distribution, a disproportionate number of ambulances and road stations to the needs and existing outdated ambulances [33]. Participants identified the improvement of medical resources and capacities as facilitators to improve the quality of relief services. Increasing human resources, equipment, ambulances and emergency medical specialists is the core of the organization’s efficiency, the timely rescue of the injured, improvement and reduction of pre-hospital mortality [26]. More of the injured will be covered by improving the quality of infrastructure and trained personnel in accidents. Using appropriate resources and adhering to protocols and guidelines, providing high-quality safe care and clinical interventions, reduces mortality by improving survival in life-threatening conditions [35].

Another obstacle to the management of relief organizations to respond is the incomplete assessment of the scene by the initial personnel present at the scene. Overcrowding at the scene has led to a lack of access to the injured and an incomplete assessment of the accident. The resources and equipment that were transferred to the scene of the accident based on an incomplete assessment were less than the required needs of the scene and disrupted the release of the injured and the provision of relief services. In this regard, Eftekhari *et al*., [29] considers one of the most important challenges of the pre-hospital system to be the lack of an accurate assessment of the injured. In order to provide optimal care, it is necessary to improve the quality of the assessment. Therefore, in order to provide optimal care, it is necessary to improve the quality of the assessment. The assessment must be very accurate in order to be able to fully identify the condition of the injured by transferring the diagnostic data of the injured to the physician of the emergency center. Determining life-threatening conditions and providing optimal care to victims require careful assessment, which is one of the most important ways to obtain vital information about victims, which is often done in a hurry. Injury assessment data is a practical guide to EMS staff decision-making at the scene of an accident. In this study, the police arrived at the scene of the accident with a delay, and in some cases, due to lack of personnel or neglect of their security role, they were not able to establish security at the scene and pave the way for irresponsible people to intervene and create disorder. According to Iranian regulations, the police are the reference in the accident scene. However, the police are not able to perform this task due to a lack of human resources, lack of technical knowledge and expertise, and lack of sufficient operational experience [28]. Bigdley’s study also showed that delays in police arrival at the scene and lack of cooperation in providing a safe environment for EMS staff is another important obstacle to timely and effective care for trauma injuries [24]. The results of this study also showed that accident scene security management as a facilitator has caused other relief organizations to provide better services. In this regard, the study of Lukumay (2019) showed that the establishment of scene security by the police has facilitated the relief services of organizations [36]. Security management at the scene of the accident, while creating a safe environment for the staff of relief organizations and the injured, as a facilitator, accelerates the provision of relief and medical services and the transfer of the injured. In cases where overcrowding disrupts the security of the scene and worsens the situation of the injured due to the intervention of people. If caregivers manage the scene, those on the scene can become an opportunity to accelerate and facilitate pre-hospital interventions [37] . Another obstacle to managing the response of relief organizations is a poor response, which has resulted from different organizational structures, goals, and lack of coordination at the scene of the accident and has led to chaos in response. The injured did not receive proper clinical care at the scene due to inconsistency and poor communication between relief organizations, lack of access to resources, incomplete assessment and weakness in providing security at the scene. Lack of evidence-based protocols and guidelines can lead to a lack of standardized clinical care and a lack of integration of communication and coordination processes in accident scene management and results in increased mortality [29]. The biggest challenge in any MCI is the ability to respond quickly and the coordination of relief organizations. To achieve the best results, it is essential to have a mechanism for evaluating, communicating, and coordinating all aspects of caring for the injured, from rescue and evacuation to definitive care [38]. Based on the results of the present study, due to the different goals and structure of Relief organizations, cooperation in response is recognized as a facilitator. For optimal management and proper response, it is necessary to provide communication infrastructure, proper information, scene security and cooperation of relief organizations at the scene of the accident. The findings of this study showed that if Relief organizations have a better understanding of each other’s resources and capacities and knowledge of using the equipment of other organizations, they will cooperate more with each other at the scene of the accident. The results of a study inconsistent with this study showed that the familiarity of Relief organizations with each other’s resources and equipment does not affect the extent of their cooperation [39]. In line with this study, Pramanik showed that the knowledge of using equipment and resources of other organizations improves the level of cooperation of organizations in the response phase and the more organizations know each other, the more they share resources and equipment and increase cooperation between organizations [40].

A number of factors have facilitated or disrupted the performance of relief organizations in MCTI. Independent relief organizations, insufficient knowledge of organizations about one other, poor response management and shortage of medical resources and capacities have led to lack of coordination in relief organizations. Challenges in inter-organizational management were information mismanagement, weakness in establishing security for the scene, and weak responsiveness. Professional interaction and better recognition of relief organizations from each other’s services and resources, establishment of a joint radio communication center, holding joint relief exercises and inter-sectoral training strengthens the coordination of relief organizations and promotes unified command in service delivery.

## Limitations and Strengths of the Study

One of the strengths of this study is using the experiences of emergency forces in four provinces, especially Qom, whose emergency forces had good experience in MCTI due to the fact that the traffic load from most provinces toward Tehran passes through Qom. Some participants did not respond to follow-up phone interviews, while others responded to the follow-up questions. The planning of relief organizations does not become operational and the MCTI management is not unified. It is suggested that the reasons for this lack of execution in the relief organizations planning are investigated. It is also recommended that the effects of centralizing the emergency bases and creating a common EOC for relief organizations on the performance be studied.

## References

[1] Lomaglio L, Ansaloni L, Catena F, Sartelli M, Coccolini F (2020). Mass Casualty Incident: Definitions and Current Reality WSES Handbook of Mass Casualties Incidents Management:.

[2] Centre DaEM, MoHaM E (2015). Reportable special events Iran: Disaster.

[3] Fararoei M, Sadat SJ, Zoladl M (2017). Epidemiology of trauma in patients admitted to an emergency ward in Yasuj. Trauma Monthly.

[4] Sadeghniiat-Haghighi K, Yazdi Z, Moradinia M, Aminian O, Esmaili A (2015). Traffic crash accidents in Tehran, Iran: Its relation with circadian rhythm of sleepiness. Chin J Traumatol.

[5] Balikuddembe JK, Ardalan A, Khorasani-Zavareh D, Nejati A, Raza O (2017). Weaknesses and capacities affecting the Prehospital emergency care for victims of road traffic incidents in the greater Kampala metropolitan area: a cross-sectional study. BMC Emerg Med.

[6] Autrey AW, Hick JL, Bramer K, Berndt J, Bundt J (2014). 3 Echo: concept of operations for early care and evacuation of victims of mass violence. Prehosp Disaster Med.

[7] Balikuddembe JK, Ardalan A, Khorasani-Zavareh D, Nejati A, Kasiima S (2017). Factors affecting the exposure, vulnerability and emergency medical service capacity for victims of road traffic incidents in Kampala Metropolitan Area: a Delphi study. BMC Emerg Med.

[8] Holgersson A (2016). Review of on-scene management of mass-casualty attacks. Journal of Human Security.

[9] Juffermans J, Bierens JJ (2010). Recurrent medical response problems during five recent disasters in the Netherlands. Prehosp Disaster Med.

[10] Dean MD, Nair SK (2014). Mass-casualty triage: Distribution of victims to multiple hospitals using the SAVE model. European Journal of Operational Research.

[11] Aitken P, Leggat P Considerations in mass casualty and disaster management. Emergency medicine: an international perspective.

[12] Anderson PD, Suter RE, Mulligan T, Bodiwala G, Razzak JA, Mock C (2012). International Federation for Emergency Medicine (IFEM) Task Force on Access and Availability of Emergency Care World Health Assembly Resolution 60 22 and its importance as a health care policy tool for improving emergency care access and availability globally. Ann Emerg Med.

[13] Pan American Health Organization ( 2019). Mass Casualty Management System.

[14] Hitti EA, El Sayed MJ, Cheaito MA, Kellermann AL, Kazzi AA (2020). Mass Casualty Management in the Emergency Department-Lessons Learned in Beirut, Lebanon-Part I. Mediterranean Journal of Emergency Medicine & Acute Care.

[15] Chaffee M (2009). Willingness of health care personnel to work in a disaster: an integrative review of the literature. Disaster Med Public Health Prep.

[16] Sadat SJ, Âli Mohammadi N, Alamdari A (2012). Phenomenological study of family and social relationships of patients with multiple sclerosis. Journal of Mazandaran University of Medical Sciences.

[17] Elo S, Kääriäinen M, Kanste O, Pölkki T, Utriainen K, Kyngäs H (2014). Qualitative content analysis: A focus on trustworthiness. SAGE open.

[18] Organization WH (2019). Hospital safety index.

[19] Burns N, Grove SK (2010). Understanding nursing research-eBook: Building an evidence-based practice.

[20] Speziale HS, Streubert HJ, Carpenter DR (2011). Qualitative research in nursing: Advancing the humanistic imperative.

[21] Raeisi AR, Mohajervatan A, Mehraein Nazdik Z (2019). Mass casualty response to mine explosion: a case report in Iran. Health in Emergencies and Disasters.

[22] Veenema TG, Boland F, Patton D, O’Connor T, Moore Z, Schneider-Firestone S (2019). Analysis of Emergency Health Care Workforce and Service Readiness for a Mass Casualty Event in the Republic of Ireland. Disaster Med Public Health Prep.

[23] Oveisi N, Farsad H, Sarikhani N, Najafi M, Mousavi AS, Zaretousi Z (2014). Studying the managers’viewpoint of east azerbaijan province about earthquake relief operation in red crescent society in 2012.

[24] Bigdeli M, Khorasani-Zavareh D, Mohammadi R (2010). Pre-hospital care time intervals among victims of road traffic injuries in Iran A cross-sectional study. BMC Public Health.

[25] Khorram-Manesh A, Plegas P, Högstedt Å, Peyravi M, Carlström E (2020). Immediate response to major incidents: defining an immediate responder!. Eur J Trauma Emerg Surg.

[26] Yu W, Lv Y, Hu C, Liu X, Chen H, Xue C (2018). Research of an emergency medical system for mass casualty incidents in Shanghai, China: a system dynamics model. Patient Prefer Adherence.

[27] Andreassen N, Borch OJ, Sydnes AK (2020). Information sharing and emergency response coordination. Safety Science.

[28] Bazeli J, Aryankhesal A, Khorasani-Zavareh D (2017). Exploring the perception of aid organizations’ staff about factors affecting management of mass casualty traffic incidents in Iran: a grounded theory study. Electron Physician.

[29] Eftekhari A, DehghaniTafti A, Nasiriani K, Hajimaghsoudi M, Fallahzadeh H, Khorasani-Zavareh D (2019). Management of Preventable Deaths due to Road Traffic Injuries in Prehospital Phase; a Qualitative Study. Arch Acad Emerg Med.

[30] Pedersen MJ, Gjerland A, Rund BR, Ekeberg Ø, Skogstad L (2016). Emergency Preparedness and Role Clarity among Rescue Workers during the Terror Attacks in Norway July 22, 2011. PLoS One.

[31] Hill B (2010). Diagnosing co-ordination problems in the emergency management response to disasters. Interacting with Computers.

[32] Zhang Y, Zheng J, Yi M, Ma H (2017). Influencing factors and mechanisms of inter-organization collaboration obstacles in emergency rescue for urban rail transit. Advances in Mechanical Engineering.

[33] Bahadori M, Khankeh HR, Zaboli R, Ravangard R, Malmir I (2017). Barriers to and Facilitators of Inter-Organizational Coordination in Response to Disasters: A Grounded Theory Approach. Disaster Med Public Health Prep.

[34] Murota T, Takeda F (2019). A Discussion on the Nation’s Command and Coordination Regarding Emergency Fire Response Teams. Journal of Disaster Research.

[35] Mehmood A, Rowther AA, Kobusingye O, Hyder AA (2018). Assessment of pre-hospital emergency medical services in low-income settings using a health systems approach. Int J Emerg Med.

[36] Lukumay GG, Outwater AH, Mkoka DA, Ndile ML, Saveman BI (2019). Traffic police officers’ experience of post-crash care to road traffic injury victims: a qualitative study in Tanzania”. BMC Emerg Med.

[37] Tannvik TD, Bakke HK, Wisborg T (2012). A systematic literature review on first aid provided by laypeople to trauma victims. Acta Anaesthesiol Scand.

[38] Born CT, Briggs SM, Ciraulo DL, Frykberg ER, Hammond JS, Hirshberg A (J Am Acad Orthop Surg. 2007). Disasters and mass casualties: I. General principles of response and management.

[39] Pramanik R, Hassel H, Tehler H (2015). Motivating factors towards willingness to contribute in collaborative tasks: A crisis cooperation perspective. ESREL 25th European Safety And Reliability Conference.

[40] Pramanik R, Ekman O, Hassel H, Tehler H (2015). Organizational adaptation in multi‐stakeholder crisis response: An experimental study. Journal of Contingencies and Crisis Management.

